# Bayesian Estimation of MSM Population Size in Côte d’Ivoire

**DOI:** 10.1080/2330443X.2018.1546634

**Published:** 2019-03-09

**Authors:** Abhirup Datta, Wenyi Lin, Amrita Rao, Daouda Diouf, Abo Kouame, Jessie K. Edwards, Le Bao, Thomas A. Louis, Stefan Baral

**Affiliations:** a Department of Biostatistics, Johns Hopkins University, Baltimore, MD; b Division of Biostatistics and Bioinformatics, University of California, San Diego, La Jolla, CA; c Department of Epidemiology, Johns Hopkins University, Baltimore, MD; d Enda-Sante, Dakar, Senegal; e Ministry of Health, Côte d’Ivoire, Abidjan, Ivory Coast; f Department of Epidemiology, University of North Carolina, Chapel Hill, Chapel Hill, NC; g Department of Statistics, Penn State University, State College, PA

**Keywords:** AIDS, Bayesian model, Côte d’Ivoire, HIV, MSM population, Small area estimation

## Abstract

Côte d’Ivoire has among the most generalized HIV epidemics in West Africa with an estimated half million people living with HIV. Across West Africa, key populations, including gay men and other men who have sex with men (MSM), are often disproportionately burdened with HIV due to specific acquisition and transmission risks. Quantifying population sizes of MSM at the subnational level is critical to ensuring evidence-based decisions regarding the scale and content of HIV prevention interventions. While survey-based direct estimates of MSM numbers are available in a few urban centers across Côte d’Ivoire, no data on MSM population size exists in other areas without any community group infrastructure to facilitate sufficient access to communities of MSM. The data are used in a Bayesian regression setup to produce estimates of the numbers of MSM in areas of Côte d’Ivoire prioritized in the HIV response. Our hierarchical model imputes missing covariates using geo-spatial information and allows for proper uncertainty quantification leading to confidence bounds for predicted MSM population size estimates. This process provided population size estimates where there are no empirical data, to guide the prioritization of further collection of empirical data on MSM and inform evidence-based scaling of HIV prevention and treatment programs for MSM across Côte d’Ivoire.

## 1. Introduction

The last five years have witnessed significant advancements in the response to HIV including universal treatment for those living with HIV, antiretroviral based pre-exposure prophylaxis to prevent HIV, and new diagnostic approaches including HIV-self testing (UNAIDS 2017). However, leveraging these strategies to achieve an AIDS-Free generation by 2030 necessitates understanding who and why people continue to acquire HIV (Beyrer et al. 2014; Stahlman et al. 2016). In concentrated epidemics, it has long been understood that the majority of HIV infections are among populations with specific acquisition and transmission risks for HIV including gay men and other men who have sex with men (MSM), sex workers, people who inject drugs (PWID), and transgender women (Beyrer et al. 2014). However, in generalized HIV epidemics, the specific proximal risks for HIV have been less studied which challenges the ability to effectively specify both the most appropriate benefactors for these new interventions as well as the number of people in need (Boily et al. 2015; Mishra et al. 2016). To address the former, there have been a number of epidemiologic and mathematical modeling studies demonstrating the importance of addressing the HIV prevention and treatment needs of key populations in the context of generalized HIV epidemics (Mishra et al. 2016). However, there remain limited empirical data on the sizes of key populations across most generalized HIV epidemic settings (Abdul-Quader, Baughman, and Hladik 2014).

Characterizing the size of key populations facilitates an understanding of the numbers of potential eligible candidates for more intensive HIV prevention interventions, the overall potential impact of those interventions when implemented at scale, and finally an improved understanding of the local HIV epidemic (Abdul-Quader, Baughman, and Hladik 2014; Holland et al. 2016). Moreover, to effectively parameterize mathematical models characterizing the modes of transmission, high quality data regarding the size, characteristics, and HIV burden among key populations are needed (UNAIDS/WHO Working Group on Global HIV/AIDS and STI Surveillance 2010). Concurrently, there has been increasing consensus on the appropriate methods for population size estimation for key populations (Quaye et al. 2015;UNAIDS/WHO Working Group on Global HIV/AIDS and STI Surveillance 2010).

While many current size estimates resulted in national estimates, less documentation in the literature has focused on subnational estimates in the majority of low and middle income settings (Tanser et al. 2014). However, it is the size estimates at the subnational level that are most often used by local ministries of health, implementing partners, and bilateral and multilateral funding agencies to guide the geographic and population prioritization of resources and efforts (Yu et al. 2014). Often empirical data collection and direct estimates of key population size have been in urban or peri-urban areas where the population densities of key populations are higher and where the infrastructure exists to facilitate sufficient access to the community (Yu et al. 2014). However, HIV prevention and treatment needs are universal, necessitating methods for estimating population size of key populations at high risk of HIV acquisition and transmission at the subnational level where there are no empirical data (Tanser et al. 2014) and in rural areas as well.

There exist a range of extrapolation methods to generate estimates at the national and subnational level. These methods differ in terms of their reliance on data, cost, and scientific rigor (Yu et al. 2014). Expert opinion involves consulting experts, including national stakeholders, technical experts, and key population groups, on how confident they are with the direct estimates and seeking their advice on how to apply these numbers to other off-sample areas. This method has low reliance on data, little cost, and relatively low scientific rigor. Simple and stratified imputation methods apply the mean from areas with direct estimates to the areas where predictions are needed. These methods have some reliance on auxiliary data and result in arguably more evidence-based rigor than relying on expert opinion alone. Less is known about other more complex methods, including regression, treating off-sample areas as a missing data problem, and using geospatial covariation or correlation to predict values, that is, small-area estimation.

In West Africa, the epidemiology of HIV has been shown to be distinct from that in Eastern and Southern Africa (Djomand, Quaye, and Sullivan 2014; Holland et al. 2016; Papworth et al. 2013). Specifically, HIV prevalence among all reproductive-aged adults has not surpassed 5% though very high burdens have been observed among key populations (Djomand, Quaye, and Sullivan 2014). The burden of HIV in Côte d’Ivoire was estimated to be 3.2% among all adults equating to an estimated half a million people living with HIV, nearly all of whom are over 15 years old. In the national strategic plan for HIV, key populations including MSM, female sex workers (FSW), and PWID have been deemed to be priority populations for HIV prevention and universal treatment for those living with HIV. However, similar to other settings, the enumeration and representative sampling of key populations has been challenged by significant stigma and even criminalization of sexual practices, orientations, and addictions (Beyrer et al. 2012). Consequently, specialized sampling strategies for key populations in these settings have included respondent-driven sampling (RDS, Heckathorn 1997), time-location sampling, prioritization for local AIDS control efforts (PLACE), and others. The majority of these studies have taken place in urban centers resulting in lim ited study of population size estimates for key populations in the majority of the country including rural, less densely populated settings (Abdul-Quader, Baughman, and Hladik 2014).

Given limited empirical data on the size of key populations in much of Côte d’Ivoire, the objective of this study was to assess the utility of small area estimation approaches to estimate the population size in 61 specific areas of Côte d’Ivoire, referred to as departments, along with proper quantification of uncertainty of those estimates. Specifically, the study aimed to utilize available direct estimates of MSM population size for a few of the departments and demographic covariates in Côte d’Ivoire in a regression setup to generate model-based estimates of population sizes of MSM for all the 61 departments. A linear regression model was used to extrapolate the percentage of MSM population to areas with no size estimate. The total male population and HIV prevalence of each department were used as covariates. Since HIV prevalence was missing for nearly half of the departments, a spatial model was deployed to impute the missing values, which then served as inputs to the extrapolation model. Finally, the extrapolation and imputation models were tied together in a Bayesian hierarchical setup, which ensured uncertainty from the imputation component was properly propagated into the final estimates and confidence intervals of MSM population sizes. To our knowledge, ours are the first probabilistic estimates of the numbers of MSM in all areas of Côte d’Ivoire prioritized in the HIV response.

In settings where public health systems are decentralized, estimates for program denominators are needed at both the national and subnational level to set actionable program targets. This is especially important if there are large regional differences in burden of disease, resources, etc. Currently subnational estimates are mostly derived from either expert opinion or simple imputation, at best. Providing more principled model-based estimates will improve the rigor upon these currently practiced methods and make assumptions explicit. The intended impact of this process is to increase uptake and use of high quality, comprehensive epidemiologic and interventional data in program planning, while increasing consensus on small area estimations of available data to guide additional data collection and programmatic efforts focused on HIV among key populations.

The rest of the article is organized as follows. In Section 2, we present the available direct estimates at a few of the departments which were inputs to our extrapolation model. We describe the data sources and survey methodology used to obtain these direct estimates. We also describe the demographic covariates that were available for the majority of the departments, to facilitate the extrapolation. In Section 3, we present details of our methodology including choice of the extrapolation model, rationale behind using total male population and HIV prevalence as covariates, the spatial model for imputing HIV prevalence, and finally, the hierarchical Bayesian model for joint estimation. In Section 4, we present results—uncertainty quantified predictions of MSM population size in all of the 61 departments, and discuss the salient findings. Finally, in Section 5, we discuss future steps to incorporate these results in ongoing and future HIV prevention programs, underscore some of the key assumptions that went into this modeling exercise, and how additional data complemented with well-principled statistical methods can lead to more improved estimates in the future.

## 2. Data Sources and Direct Estimates

Direct estimates refer to estimates of population size for a specified geographical area using empirical data from survey sampling studies. In contrast, the goal of this article is to generate indirect extrapolated estimates for areas with no direct data on MSM population size. In this section, we provide a description of the direct estimates that were available at few departments in Côte d’Ivoire, along with a description of the departments where we want to predict MSM population sizes, and the covariates available to aid in the extrapolation.

### 2.1. Data Sources

Information on MSM population size was available from a number of sources at five departments of Côte d’Ivoire: Abidjan, Agboville, Bouake, Gagnoa, and Yamoussoukro. Counts of MSM were taken from programme data for NGO membership, service provision, and social event attendance. Specifically, the following counts were available for the five departments:

#### Unique object:

One method to evaluate population size makes use of unique object distribution. Objects are handed out to members of the population. The number of MSM who have received a unique object can be obtained from the log of how many objects were distributed. The unique objects serve the purpose of tagging or marking members of the key population. In a subsequent survey, participants will be asked whether they had received a unique object before, which will help to estimate the population size. More details on this are provided later in this section and Section 2.2.

#### Service:

A second method involves using clinic or service provider total counts. Counts of a population using a particular service, in conjunction with a second source of information, can provide information about the population size as will be detailed in Section 2.2. In this case, the total number of MSM attending services at “Clinique de Confiance” was captured from program logs.

#### NGO membership:

Similarly, a record of the members of an organization is a useful data source about the population. The total number of MSM belonging to NGOs was also captured from program logs.

#### Social event:

If a special event is planned by the MSM community for MSM members, a list of attendees can constitute the first of the two data sources needed to estimate the population size. In this case, data on the total number of MSM who attended the social event “evening GNARA” was available. Not all five departments had records for all the sources. Some were missing as detailed in Table 1.

In addition to these count data, a second independent source of information on the MSM population is needed to estimate the population size. For the five departments, there was a representative survey in which MSM were recruited through RDS (Heckathorn 1997), a strategy employed when individuals in the target population are hard-to-reach and when no known sampling frame exists. Methods for RDS have been described and compared previously (White et al. 2012; Stahlman et al. 2016; Wirtz et al. 2016). Individuals were purposively asked questions about their involvement in the aforementioned services which provides counts of MSM. As one example, for the service multiplier method, participants were asked if they had ever received services from “Clinique de Confiance” (see Appendix A for the list of questions asked for each source category). The binary responses to these questions, combined with the respective total counts obtained from the program logs helped to derive direct estimates for MSM population using the multiplier method as outlined in Section 2.2.

### 2.2. Direct Estimates via the Multipler Method

Multiplier methods are a standard approach to directly estimate the size of a subpopulation. A first data source provides a total count (*T*) of the subpopulation who participate in a certain event or activity, and a second independent data source provides an estimate of the proportion ($\hat{p}$) of that subpopulation who are associated with the aforementioned activity. The basis for this method rests on the assumption that the proportion of individuals in the subpopulation who appear at a specific institution (which provides the first source) is equal to the proportion who appear at that same institution among the second source. For example, the proportion of all MSM who are currently registered in an NGO is assumed to be equal to the proportion of the members of the same NGO among the survey participants. The population size estimate *N* then satisfies $T/N\;=\hat{p}$ and is estimated by $N\;=\;T/\hat{p}$.

For each of these five departments, size estimates were available to us which had been previously generated through the use of various multiplier methods. The multiplier method is commonly named after the data source providing the count of MSM participating in that event, that is, unique object multiplier, NGO membership multiplier, service multiplier, and social event multiplier. The RDS survey is the second independent data source which contained binary responses about involvement of the survey participants for each of the four events/activity listed as the first source.

Since RDS is a network-based sampling approach, the Volz-Heckathorn estimator (Volz and Heckathorn 2008) uses the personal network sizes of the participants to obtain estimates of characteristics of the population. If *b*_*i*_,_NGO_ denotes the binary indicator for the *i*th survey participant being a member of an NGO, and *d*_*i*_ denotes the reported network size of that individual, then the Volz-Heckathorn estimator (also commonly referred to as the RDS II estimator) of ${\hat{p}}_{\text{NGO}}$ —the proportion of MSM who are NGO members is:

$${\hat{p}}_{\text{NGO}}=\frac{\sum_{i}\frac{b_{i,\text{NGO}}}{d_{i}}}{\sum_{i}\frac{1}{d_{i}}}.$$

Subsequently, using the total count *T*_NGO_ of MSM registered for that NGO, the NGO multiplier estimate of MSM population size is given by $T_{\text{NGO}}/{\hat{p}}_{\text{NGO}}.$ Similarly, other multiplier based size estimates can be derived based on the respective count of other first sources and the corresponding binary responses in the RDS.

Table 1 presents the multiplier based direct estimates of MSM in the age group of 18–29 years for the five areas. The column names indicate which data source was used as the first source in the multiplier method, with the RDS survey being the second source. Confidence intervals for the Volz–Heckathorn estimators of the proportions were calculated based on Salganik (2006) which were then used to calculate the confidence intervals of the population size estimates, as reported in Table 1. Note that not all survey methods were implemented in all areas. In Abidjan, there were two NGos where we had access to the total count of members. On the other hand, Agboville does not have a service multiplier based estimate and Gagnoa does not have an estimate from NGO membership.

The direct estimates presented in Table 1 were already available to us and have been used in the web-report (http://www.endasante.ci/images/rapport/rapport_IBBS_MSM_2015_2016.pdf). Our main goal is to use these direct estimates in a regression model to produce the estimates of MSM population size in areas with no data. Hence, we did not pursue more methodological developments to improve direct estimates using multiplier methods based on an RDS, but rather focused on using the direct estimates derived using certain assumptions as inputs to the extrapolation model.

Nonetheless, we note that several assumptions are used in the calculation of direct estimates in Table 1. First, at the heart of the multiplier method is the assumption that the first and second sources of data are independent, which may not be the case. We discuss this more later in Section 5 in the context of the results. Also, even if the four different sources used as the first data source in the multiplier method are each independent of the RDS, they may not be independent of each other. Size estimation methods based on multiple capture–recapture exist (Castledine 1981). However, they usually require the total number of subjects that participated in at least one of the independent surveys. This information is not available to us as the overlaps between the four sources are unknown. Hence, we could not use joint multiplier method in its most basic implementation. However, since we have multiple areas here, there might be alternate ways of modeling this dependency via jointly modeling all the areas under certain assumptions. We did not pursue them here. Finally, because those sampled were 90% from the age group 18–29, all population size estimates produced in this manuscript are for this age group.

### 2.3. Prediction Areas and Covariates

In total, there were 61 prediction areas that were selected to coincide with PEPFAR organizational units (OUs) to provide evidence-based estimates for targeted prevention, care and treatment programs and to inform country operational processes. These PEPFAR departments also roughly correspond with the official department-level administrative divisions in Côte d’Ivoire.

Covariates were selected based on relevance to the prediction model (as will be detailed in Section 3.2) and availability of quality data at the appropriate administrative division (department) level. Data for population density, density change and male population were obtained from publicly available data published by the Institut National de la Statistique, Republique de Côte d’Ivoire. Data for HIV prevalence was obtained from a UNAIDS report on subnational estimates of HIV prevalence in Côte d’Ivoire (http://www.unaids.org/sites/default/files/media_asset/2014_subnationalestimatessurvey_Cotedivoire_en.pdf).

There was no department-level age-stratified, sex-stratified data. Therefore, we assumed a constant age and sex distribution across all departments: 55% of total male population for each of the departments/area seats is in the age group 18–29. Also, for Abidjan, close to 90% of our sample reported being from either Abobo, Cocody, Marcory, Triechville, or Youpougon. This is just five communes out of the total ten in Abidjan. We therefore considered our sample to better represent these five communes of Abidjan rather than the whole city. The total male (15–49) population for these communes was 842,551 rather than 1,286,750 for the whole city, and for men 18–29 it was 368,097 rather than 562,160. We also assumed that the age-sex distribution was the same across all communes.

Additionally, we used estimated population density from the Landscan database (http://web.ornl.gov/sci/landscan/) based on night light data. The night light data is from Defense Meteorological Satellite Program (DMSP) Operational Linescan System (OLS) which detects nighttime lights from satellite imagery. Landscan provided estimated population size over 1 km × 1 km grid cells. For each of the 61 prediction areas, the population estimates were averaged over a 25 km^2^ radius centered on the area to obtain the average population density for the areas.

## 3. Methods

In this section, we describe our methodology. We used the direct estimates provided in Table 1 for the five departments to train a regression model for predicting the MSM population size based on the covariates, and subsequently used this model to extrapolate to all other areas. Although the direct estimates are not raw data but are statistical end-products from the multiplier method, we treat them as “data” for the extrapolation model. Hence, the extrapolation model in this section can be viewed as the second step of a two-step approach where the first step is to calculate the direct estimates as detailed in Section 2.2. Since our main objective is the prediction of MSM population size for the areas without direct data, we will plug-in the available direct estimates as “pseudo data” for the regression model and use the confidence intervals for the direct estimates to specify the heteroscedastic variances in the model. In Section 5, we expand on why we chose this two-step method and the difficulties of implementing a comprehensive method that uses the actual raw data, that is, counts and individual survey responses.

### 3.1. Linear Model

Most of our modeling choices are guided by the extremely small sample size (a total of 19 datapoints from five departments), which proscribed the use of complex models involving many parameters. The covariates described in Section 2 were area specific. Hence, although there were 19 datapoints, there were only five unique sets of covariate values, one for each area. This impeded exploring nonlinear models linking the MSM population size to the covariates, and confined us to the parsimony of the linear model. For the *i*th area, let *N*_*i*_ denote the total male population in the age group of 18–29 years, *x*_*i*_ denote the set of demographic covariates and *n*_*ij*_ denotes the direct estimate obtained from the *j*th method. A natural choice for modeling the population size would have been a generalized linear model (GLM) *n*_*ij*_ ~ Binomial(*N*_*i*_,*p*_*i*_) where $p_{i}=\;\text{logit}\;\left(x_{i}^{T}\beta \right)=\frac{\exp\left(x_{i}^{\prime }\beta \right)}{1+\exp\left(x_{i}^{\prime }\beta \right)}$ However, note that not all the direct estimates are equally precise. For example, we observe in Table 1 that the NGO membership-based estimate of MSM population in Bouake differs by an order of magnitude from the other three estimates for the same area. The confidence interval for this estimate is also very wide suggesting low precision of the estimate. Since it is less clear howto incorporate information from the confidence intervals in a binomial GLM setup, we used a suitable transormation of the size estimates and worked in the linear regression paradigm. This allowed us to leverage the confidence bounds of the direct estimates in Table 1 in a linear regression setup via heteroscedastic errors.

We used log-transformation of the fraction of total 18–29 male population who are MSM as the response and specified the linear regression model as

(1) $$y_{ij}\overset{\;\text{ind}\;}{\sim }N\left(x_{i}^{T}\beta ,\tau_{ij}^{2}\right),\;\text{where}\;y_{ij}=\log\left(n_{ij}/N_{i}\right).$$

We used the confidence intervals provided in Table 1 to specify the heteroscedastic errors < $\tau_{ij}^{2}$. Specifically, if (*n*_*ij*_._*l*_, *n*_*ij*_._*u*_) denotes the 95% confidence interval of *n*_*ij*_ in Table 1, then (*y*_*ij*_._*l*_,*y*_*ij*_._*u*_) is a 95% confidence interval for *y*_*ij*_, where $y_{ij.l}=\log\left(n_{ij.l}/N_{i}\right)$ and $y_{ij.u}=\log\left(n_{ij.u}/N_{i}\right)$. Since the variance of a normal random variable is proportional to the square of the width of the 95% inter-quantile range, we specified $\tau_{ij}^{2}=\tau^{2}{\left(y_{ij\cdot u}-y_{ijl}\right)}^{2}$. This ensures that more uncertain estimates with very wide confidence bounds are given less weight in the model than the ones with narrow confidence intervals providing more precise information.

Regression models for small area estimation often include area specific random effects to improve estimation (Fay and Herriot 1979). However, Datta, Hall, and Mandal (2011) argued that when the number of areas is small, the simpler model without random effects often performs better. Owing to the very small sample size of the dataset, we decided against introducing area specific random effects as it involves additional parameters.

Finally, we used a log-transformation to define the *y*_*ij*_s in 1) although a logit transformation is more natural, as *n*_*ij*_*/N*_*i*_ is a proportion. In the dataset, the proportions *n*_*ij*_*/N*_*i*_ are typically very small (80% are smaller than 0.05). So the two transformations yield very similar *y*_*ij*_s. Furthermore, as we discuss in Section 3.2, the log transformation is more interpretable in our final model which includes log(*N*_*i*_) as one of the covariates.

### 3.2. Covariate Selection

Given the limited data size, we wanted to select only one or two most relevant covariates from the five available—male population, population density, density change, HIV prevalence, and Landscan density. We kept HIV prevalence in the model as we expected areas where there are more MSM to be areas with high HIV prevalence, as MSM are disproportionately affected by HIV given the biology of HIV transmission.

We conducted model selection using leave-one-out cross-validation to choose the second covariate (alongside HIV prevalence). Hence, there were five candidate models—one with HIV prevalence as the only predictor, and four others with HIV prevalence and each of the four other covariates as the second predictor. For each of the five models, we left all the direct estimates from one area out and fitted the model using the remaining areas to predict the MSM population size for the left-out area. We repeated this for all the five areas. If *n*_*i*_ denotes the mean of the direct estimates for the *i*th area and ${\hat{n}}_{i}{,}_{M}$ denotes the estimate of MSM population for the *i*th area using model *M* and all the direct estimates except those for the *i*th area, the leave-one-out cross validated mean square error (MSE_LOOCV_) for the model *M* is given by $\sum_{i=1}^{5}{\left(n_{i}-{\hat{n}}_{i,M}\right)}^{2}$.

**Figure 1.**
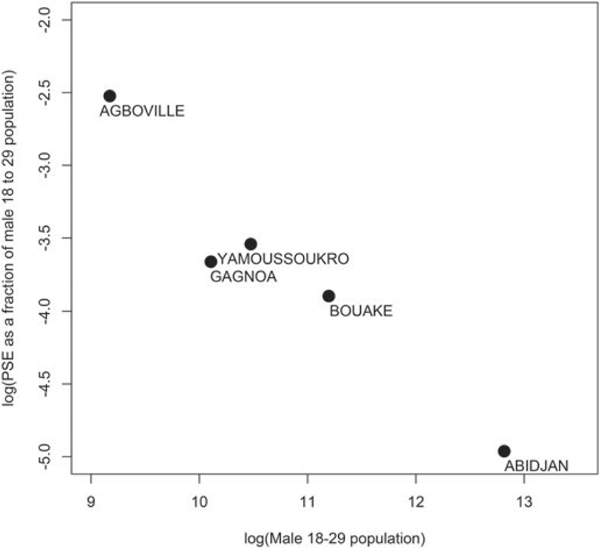
Negative correlation in log–log scale between MSM proportion and total male population of age 18–29.

Table 2 provides the LOOCV based mean square error for the five models. The model with log male population and HIV prevalence as covariates had the lowest MSE. This result was surprising to us as the total male population in the age group (*N*_*i*_) has already been used to define *y*_*ij*_’s in (1). However, Figure 1 reveals that *y*_*ij*_’s have a very strong negative correlation with log(*N*_*i*_)’s. In fact, each of the four population based covariates considered in Table 2 were negatively correlated with *y*_*ij*_’ s, with log (*N*_*i*_) exhibiting the strongest negative correlation.

Initially, this negative correlation seems counter-intuitive given a rural-to-urban migration. One conjecture that explains this trend is that in large urban centers the MSM community grows at a slower rate than the overall population even if the absolute numbers of MSM is higher in those urban areas. For example, if the numbers of MSM population for the *i*th city (*n*_*i*_) grows at a rate proportional to $N_{i}^{\gamma }$ for some *γ <* 1, then although the absolute MSM numbers are positively correlated with total population (cov(log(*n*_*i*_), log(*N*_*i*_)) α *γ* > 0), the proportion of MSM is negatively correlated with the total population (cov(log(*n*_*i*_/*N*_*i*_), log(*N*_*i*_)) α *γ* – 1 < 0). We provide additional discussion on alternate explanations for this relationship in Section 5. Including log(*N*_*i*_) as a covariate, in turn, prompted us to use a log-transformation of MSM proportions (and not logit transformation) for the linear regression model, as it imparted the nice interpretability about the relative growth rates of the MSM population and the total population mentioned above.

The final linear regression model was $y_{ij}=\beta_{0}+\beta_{1}\log\left(N_{i}\right)+\beta_{2}H_{i}+\epsilon_{ij}$ where *H*_*i*_ denote the HIV prevalence for the *i*th area.

### 3.3. Spatial Model for HIV Prevalence

HIV prevalence data were missing for about 50% (30 out of 61) of the locations where we want to predict MSM population, and we need to impute the missing values. A simple choice for imputation would be to use the average of the observed values. However, exploratory analysis suggested that HIV prevalences in nearby areas were generally more similar, a spatial correlation that was confirmed by an empirical variogram. The variogram is an effective tool for detecting spatial structure. It plots pairwise squared data differences, averaged over bins of spatial distances, as a function of the distance.

**Figure 2.**
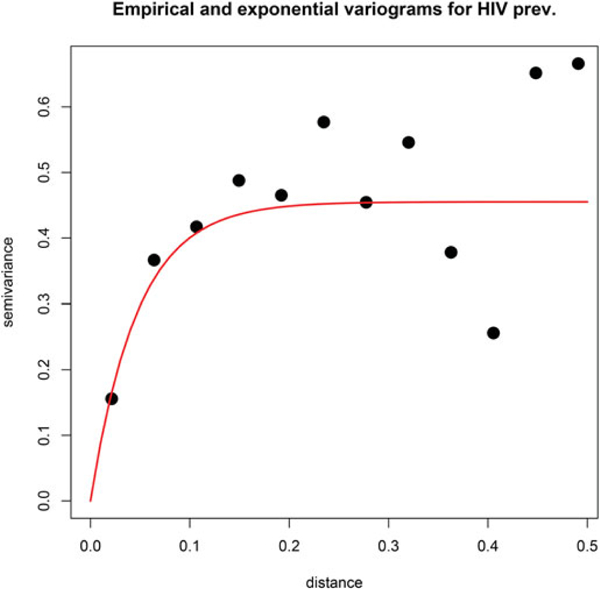
Empirical (black dots) and exponential covariance Gaussian process (red line) variogram for HIV prevalence.

Formally, let *H*(*s*_*i*_) *= H*_*i*_ denotes the HIV prevalence at an area with geographical co-ordinates *s*_*i*_. Let *B*_*k*_ denote the collection of pairs (*i*, *j*) such that *I*_*k*–1_ ≤ ||*s*_*i*_ – *s*_*j*_|| ≤ *I*_*k*_ with || • || being the Euclidean distance. Then, the empirical variogram for the mid-point of *m*_*k*_ of the interval (*I*_*k*–1_*, I*_*k*_) is given by

$$v\left(m_{k}\right)=\frac{\sum_{(i,j)\in B_{k}}(H\left(s_{i}\right)-H{\left(s_{j}\right)}^{2}}{\sum_{(i,j)\in B_{k}}1},$$

and is interpreted as the average squared data difference for HIV prevalences for areas separated by approximately *m*_*k*_ distance. A variogram with small values for low *m*_*k*_ (high positive correlation) and higher values for large *m*_*k*_ (low positive correlation) indicates spatial dependence that decreased with distance. Figure 2, which plots the empirical variogram, confirms this trend.

A Gaussian process (GP) is a popular tool for modeling data whose empirical variograms indicates spatial structure, such as that in Figure 2. Consequently, we modeled *H*(*s*) as a GP with a constant mean and an exponential covariance function. If *H*(*S*) denotes the vector formed by stacking up the HIV prevalence data for the set of areas *S* that have data on HIV prevalence, then the GP specification uses the multivariate Gaussian distribution $H(s)\sim N(\mu 1,\Sigma )\;\text{where}\;\Sigma =\sigma^{2}\exp{\left(-\phi \left\|s_{i}-s_{j}\right\|\right)}_{s_{i},s_{j}\in S}$. We plot in Figure 2 the model-based variogram corresponding to this exponential GP. This fits the exponential variogram σ^2^(1 – exp(–*ϕm*_*k*_)) (red curve) to the points *v*(*m*_*k*_) from the empirical variogram. The fit documents the utility of using GP for modeling HIV prevalence. Using the GP model, the prevalence data at *S* is used to estimate the parameters (*μ*, σ^2^, *ϕ*). Subsequently, the prevalence at any new location *s* is given by

(2) $$[H(s)|H(S)]\sim N\left(v{\left(s\right)}^{T}\Sigma^{-1}H(S),\sigma^{2}-v{\left(s\right)}^{T}\Sigma^{-1}v(s)\right),$$

where $v(s)=\sigma^{2}{\left(\exp-\phi \left\|s-s_{i}\right\|\right)}_{s_{i}\in S}$. The conditional distribution in (2) used to predict HIV prevalence at any location *s* is commonly referred to as “kriging” in the geostatistics literature. We refer the reader to the books by Cressie and Wikle (2011) and Banerjee, Carlin, and Gelfand (2014) for more details on variograms, spatial GP models and kriging.

We compared the predictive performance of the spatial model with simple mean imputation for HIV prevalence using leave-one-out cross-validation. For the cross-validation, we used the kriging equations in (2) to impute the HIV prevalence at each left-out area, based on parameter estimates using data from the remaining areas. For comparison, we used the mean HIV prevalence of the in-sample data to predict at the left-out area. The leave-one-out MSE for the spatial model (MSE = 0.37) was around 20% better than for the mean imputation (MSE = 0.48). Hence, we used the spatial GP model for imputing the missing HIV prevalence data.

### 3.4. Hierarchical Bayesian Modeling

Obtaining meaningful confidence bounds for the predicted MSM population is critical. The uncertainty of the regression parameters and especially the spatial parameters are often ignored. Furthermore, for areas with missing HIV prevalence data, the kriging estimates in Equation (2) are accompanied by the kriging variances which can be large if the location is far from the data locations. Ignoring this source of uncertainty can lead to prediction bounds that are too narrow. In a frequentist approach, it is unclear how to use the kriging variance when the imputed HIV prevalence will be used as a covariate to predict MSM population size. However, we can seamlessly integrate this multistage procedure into a hierarchical Bayesian model that allows for proper propagation of uncertainties associated with all inferences for all model components.

To do so, let *S* denote the set of locations where HIV data are available. Also, for any location *s,* let *N*(*s*) and *H*(*s*), respectively, denote the male population in the 18–29 age interval and the HIV prevalence. Define $y_{j}\left(s_{i}\right)=y_{ij},w_{ij}={\left(y_{ij,u}-y_{ij,l}\right)}^{2}$, and $\beta ={\left(\beta_{0},\beta_{1},\beta_{2}\right)}^{\prime }$, and the full specification of the hierarchical model is given by:

(3) $$\begin{array}{l} \prod_{i=1}^{5}\prod_{i}N\left(y_{j}\left(s_{i}\right)|\beta_{0}+\beta_{1}\log\left\{N\left(s_{i}\right)\right\}+\beta_{2}H\left(s_{i}\right),\tau^{2}w_{ij}\right)\times \\ N\left(H(S)|\mu 1,\Sigma \left(\sigma^{2},\phi \right)\right)\times \\ N\left(\beta |0,10^{6}I\right)\times N\left(\mu |0,10^{6}\right)\times \;\text{Unif}\;(\phi |0,10)\times \\ \;\text{Gamma}\;\left(1/\tau^{2}|0.01,0.01\right)\times \;\text{Gamma}\;\left(1/\sigma^{2}|2,1\right). \end{array}$$

The top row of (Equation 3) is the log-normal regression model for the MSM percentages, the middle row is the spatial GP model for HIV imputation and the bottom two rows are the parameter priors. Gamma(*a*, *b*) denotes the Gamma distribution with shape parameter *a* and rate parameter *b* and Unif(*a*, *b*) is the uniform distribution on (*a, b*). We use the *Nimble* package in R (https:\\r-nimble.org) to generate 30,000 MCMC samples from this model, the first 15,000 of which is discarded as burn-in. The posterior estimates for all the parameters are provided in Table 3.

We observe, from the estimate of *β*_1_, that there is strong negative association between *y*_*ij*_ and log(*N*_*i*_) whichwehavediscussed in Section 3.2. The association of MSM population size with HIV prevalence is relatively weak as the credible interval of *β*_2_ covers zero. The estimates of the spatial parameters indicate a strong spatial dependence in HIV prevalence, previously insinuated by the variograms in Figure 2.

### 3.5. Prediction

We used composition sampling (Chib 2001) to obtain posterior predictive distributions of MSM population size at a new location. If *s* ∈ *S*, then the predictive distribution is given by the samples $\{y_{\text{new}}^{\left(m\right)}(s)=\beta_{0}^{\left(m\right)}+\beta_{1}^{\left(m\right)}\log\{N(s)\}+\beta_{2}^{\left(m\right)}H(s)|m=1,2,\ldots ,M\}$ where $\left\{\beta_{i}^{\left(m\right)}|m=1,2,\ldots ,M\right\}$ denotes the MCMC samples from posterior distribution of *β*_*i*_. For locations outside *S* with no HIV prevalence data, the posterior distribution of *H*(*s*) is given by

$$\int p\left\{H(s)|H(S),\mu ,\sigma^{2},\phi \right\}p\left\{\mu ,\sigma^{2},\phi |H(S)\right\}d\mu d\sigma^{2}d\phi .$$

This is effectively accomplished using the samples {*μ*^(*m*)^, (σ^2^)^(*m*)^, *ϕ*^(*m*)^} to generate *H*(*s*) *| H*(*S*) via the kriging equation in (2). Subsequently, the samples $\{y_{\text{new}}^{\left(m\right)}(s)=\beta_{0}^{\left(m\right)}+\beta_{1}^{\left(m\right)}\log\{N(s)\}+\beta_{2}^{\left(m\right)}H^{\left(m\right)}(s)|m=1,2,\ldots ,M\}$ represent the posterior predictive distribution for MSM population size at those locations.

By necessity, we had to train the linear regression model on a very limited set of predictor values, based on just five unique data points. It is difficult to assess a priori whether we can extrapolate this linear relationship to other areas with significantly different demographics. We observed that for some areas with very low population, the predicted MSM population percentage was noticeably high. Further investigation into this reveals that the minimum total male population among the five departments with direct estimates corresponds to the 36th percentile of the empirical distribution of total male population among all the 61 areas. Hence, the training data corresponds to larger areas with greater population and does not inform much about the regression relationship in areas where the population is very low. This, combined with the strong negative value of *β*_1_ in Table 3 results in such high estimated MSM fractions.

For a heuristic remedy, we assumed that the negative relationship flattens out below a certain population threshold. We truncated the total male population at the 10% quantile of the empirical distribution and used these thresholded values for prediction. While this is ad hoc, more formal methods like estimating the truncation point based on the data will always truncate within the data values, whereas replacing a linear regression with a general monotonic function will involve more parameters and hence is infeasible for our small dataset. Our truncation did not affect parameter estimation because all the total population values for the training data are above the truncation level. This issue is less severe for HIV prevalence as the observed values for the five departments better represented the empirical distribution of HIV prevalence. Since it also has a much weaker association with population size of MSM, we did not truncate the HIV prevalence values.

## 4. Results

We discuss the quality of HIV imputation in Section 4.1 and then in Section 4.2 we summarize predictions of MSM population size estimates and discuss the impact of different factors on the resulting predictions and prediction variances.

### 4.1. Imputed HIV Prevalences

**Figure 3.**
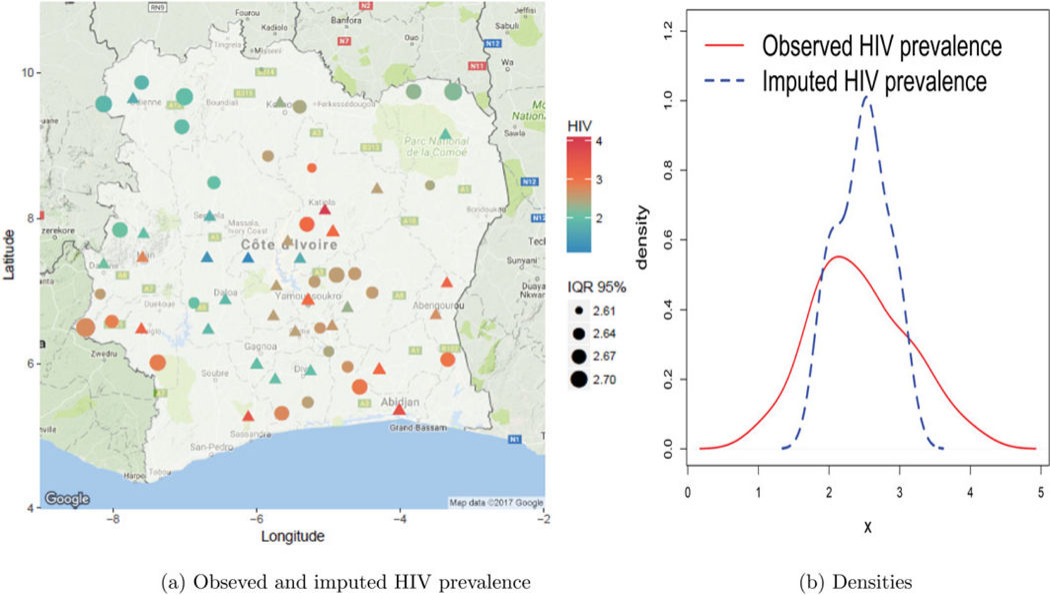
HIV imputation: plots of (a) predicted and observed HIV, where Δ represents observed data, • denotes predictions, and IQR 95% is the 95% inter-quantile range of predictions, that is, width of the 95% credible interval; and (b) overall densities of observed and imputed HIV prevalences

Figure 3(a) represents the observed and imputed HIV prevalences along with the associated uncertainties (inter-quantile ranges) of the imputed prevalence. We see that the imputed HIV prevalences are roughly similar to the observed ones in nearby departments. Overall, there is no distinct trend suggesting that imputed HIV prevalences were generally higher or lower than observed ones. To confirm that indeed there is no general bias in HIV imputation, in Figure 3(b) we plot the densities of observed and imputed HIV prevalences. The median, first, and third quartiles for observed prevalence are (2.50, 2.00, 2.95) whereas for imputed prevalence they are (2.50, 2.20, 2.70). The densities are concentrated in a similar area with the imputed prevalence having narrower tails on either side. Generally, the imputed prevalences do not exhibit any bias.

### 4.2. Size Estimates

**Figure 4.**
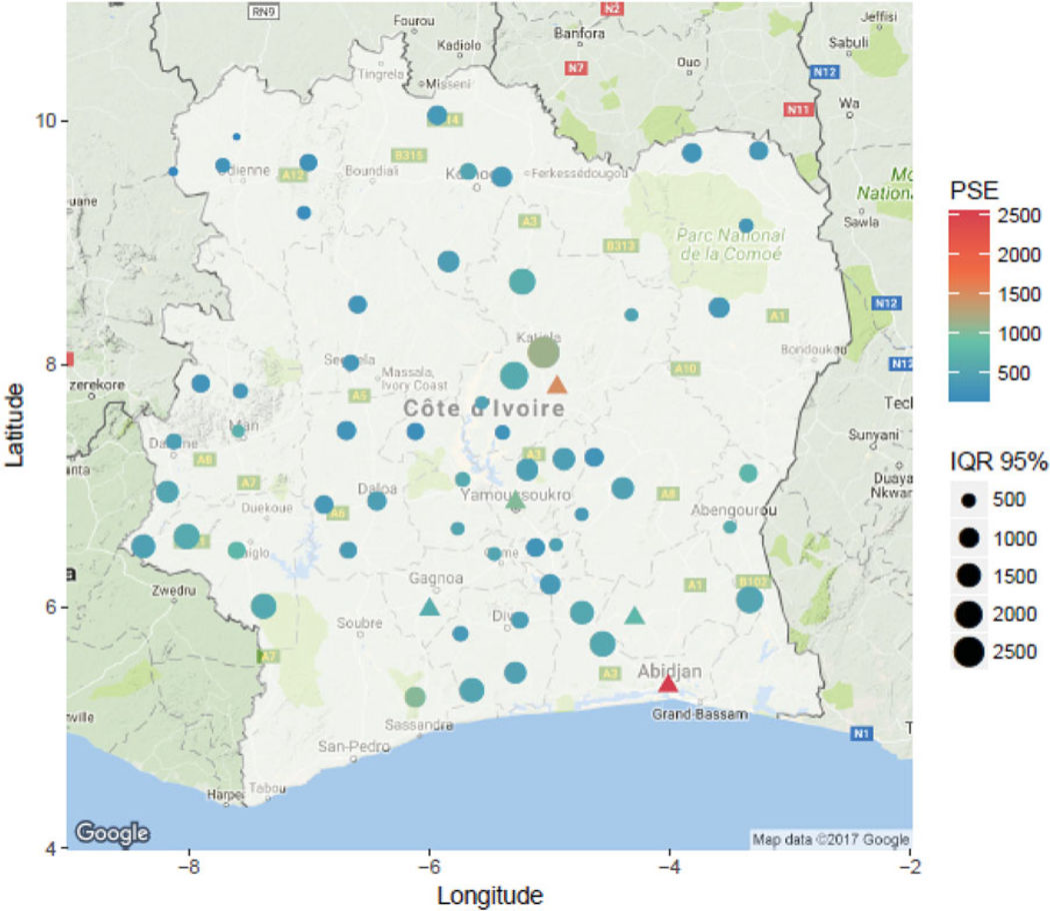
Predicted and observed MSM population. Δ represents observed data, • denotes predictions and IQR 95% is the 95% Inter-quantile range of predictions, that is, width of the 95% credible interval.

Figure 4 presents the uncertainty quantified predictions of MSM population size. The full set of predictions along with confidence intervals for all the 61 departments are presented in Table B1 in Appendix B. In terms of absolute numbers, Abidjan has by far the highest predicted MSM population size though it has one of the lowest percentages. Both occurrences are due to the massive population of Abidjan. Outside of the five data areas, Katiola and Sassandra has the highest predicted MSM population size whereas some areas have predicted numbers as low as 100. For the areas without direct estimates, the predicted MSM population percentage typically varied between as low as 0.5% to around 10%. The highest MSM percentages are predicted in Katiola, Kouassi-kouassikro, and Bettie. However, these areas also had the widest credible intervals indicating the large uncertainties associated with the predictions.

**Figure 5.**
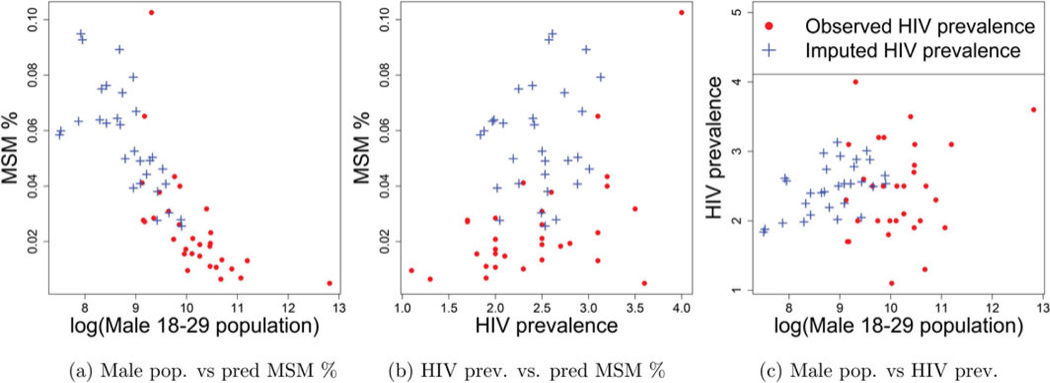
Pairwise scatterplots of the predicted MSM %, male 18–29 population and HIV prevalence.

To understand how the predicted MSM population size varied with the total population and HIV prevalence, we plotted pairwise scatterplots in Figure 5. We see from Figure 5(a) that there is strong negative correlation between the predicted MSM percentages and the total male population. This is expected in the context of the trend observed in Figure 1. Figure 5(b) reveals that there is no such overall strong trend in MSM proportions with respect to HIV prevalence. However, generally, the predicted MSM proportions seems to be higher for areas with missing HIV. The scatterplot in Figure 5(c) shows that a lot of the areas with missing HIV prevalence have low population suggesting that the high predicted MSM numbers in areas with missing HIV are not an artifact of the imputation model, but a consequence of the negative association of MSM proportion with total male population size.

**Figure 6.**
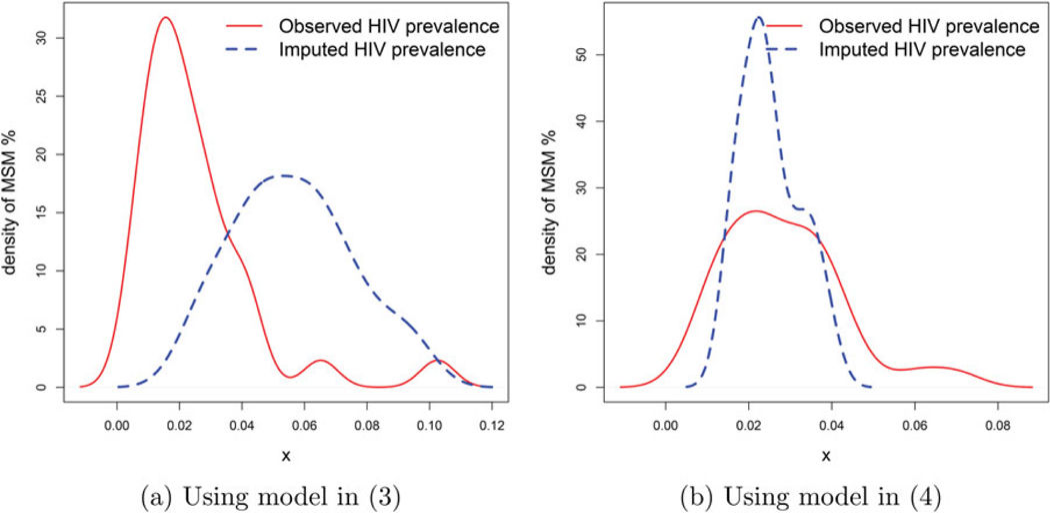
Densities of the predicted MSM % for departments with observed or imputed HIV prevalence.

To confirm that low population, and not imputed HIV, is the driver for high predicted MSM percentage, in Figure 6 we compare the densities of predicted MSM from our model with the following hierarchical model having only HIV prevalence as the predictor:

(4) $$\begin{array}{l} \prod_{i=1}^{5}\prod_{j}N\left(y_{j}\left(s_{i}\right)|\beta_{0}+\beta_{2}H\left(s_{i}\right),\tau^{2}w_{ij}\right)\\ \times N\left(H(S)|\mu 1,\Sigma \left(\sigma^{2},\phi \right)\right)\\ \times N\left(\beta |0,10^{6}I\right)\times N\left(\mu |0,10^{6}\right)\times \;\text{Unif}\;(\phi |0,10)\\ \times \;\text{Gamma}\;\left(1/\tau^{2}|0.01,0.01\right)\times \;\text{Gamma}\;\left(1/\sigma^{2}|2,1\right). \end{array}$$

Figure 6(a) shows how our model-based predicted MSM proportions are generally higher for the departments with imputed HIV prevalence. However, this is not the case for predictions based on the model from Section 3.5 where the densities for departments with or without HIV prevalence are concentrated around the same area. Both models use the same spatial imputation method for HIV prevalence, with the only difference being setting aside the total MSM population covariate in the model proposed in Section (3.5), confirming that imputing HIV prevalence is not causing high predicted MSM proportions; rather the difference is due to the strong negative association of MSM proportion with total male population.

**Figure 7.**
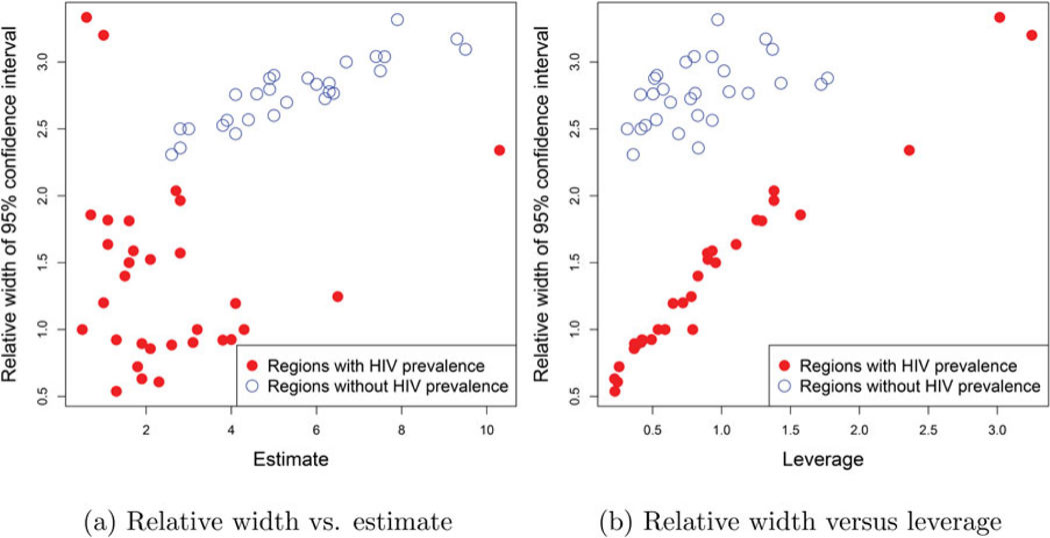
Impact of imputing HIV on uncertainty: Plots of (a) relative 95% CI widths versus estimates and (b) relative 95% CI widths versus the leverages. Red and blue dots correspond to, respectively, areas with and without HIV prevalence

Finally, we investigate the effect on the imputation of prediction uncertainties. Figure 7 demonstrates the impact of HIV imputation on the prediction uncertainties. Since the variance and width of inter-quantile ranges of log-normal distribution are proportional to the mean, we use relative width (ratio of the 95% confidence interval width to the estimate) as a more meaningful measure of uncertainty. In Figure 7(a) we plot predicted MSM population percentage against relative width. We observe that the relative width was in general larger for locations with missing HIV prevalence data. This is expected as the Bayesian model properly propagates the uncertainty associated with the imputation of HIV prevalence in the final predictions and is reflected in the CI widths. In Figure 7(b) we plot the relative width against leverages $(x_{i}^{⊤}{\left(X^{⊤}X\right)}^{-1}x_{i}$, where *X* is the design matrix and *x*_*i*_ denotes the covariate vector for the *i*th area) for each area. For areas with HIV prevalence data, relative width increases with the leverage as expected indicating that predictions for areas with covariates values distant from those of any of the observed areas are accompanied with larger uncertainty. Among areas with missing HIV prevalence, this trend was less prominent due to the added component in the uncertainty from the imputation.

## 5. Discussion

We have reported fully Bayesian predictions combined with appropriate credible bounds for the population size of MSM in the 61 areas prioritized for HIV prevention and treatment services across Côte d’Ivoire. Our analysis was based on several assumptions and the results need to be interpreted carefully.

The direct estimates calculated for the five departments assumed that the enlistment in any of the four services leading to the first source of data in the multiplier method is independent of participation in the RDS survey. If this independence assumption fails, biases are introduced into the direct estimates. We conjectured that the negative association between the direct estimates of MSM percentages and the total male population in Figure 1 is due to relative rural to urban immigration rates of MSM and the broader population. While rural to urban migration is common for MSM, the political unrest in 2002 and 2010–2011 in Côte d’Ivoire was associated with significant population-wide migration to Abidjan which may have changed population dynamics in the city, leading to such an occurrence. Alternatively, it may be possible that the independence assumption in deriving the direct estimates was violated and that smaller departments have a higher extent of overlap between the surveys. In that case, these direct estimates, assuming independence, are biased upward for smaller departments and consequently the MSM population sizes at areas with low populations are also being overestimated. A third explanation for the negative correlation would be that in urban areas a higher proportion of MSM are not accounted for in the survey. While all three reasons are conceivable, the first is a feature of MSM population dynamics while the second and third are sampling issues.

Without additional data, it is not feasible to decide among these scenarios. However, internet-based surveys may facilitate learning more about the numbers of MSM in more stigmatizing settings. In rural areas where MSM populations are small and social stigma is high, risks involved in participating in conventional face-to-face surveys may lead to significant overlap among the participants of multiple surveys. Anonymity offered by the internet may help to better sample such stigmatized populations. There is a growing body of literature on using the internet to survey MSM (Bowen, Williams, and Horvath 2004; Stromdahl et al. 2015; Buckingham et al. 2017) and direct estimates from such internet-based surveys may be more reliable in terms of conforming to the sampling assumptions. If such a survey also reveals similar trends of high percentages of MSM in low population areas, it will confirm that this is more a feature of the MSM demographics in the country and not a sampling issue.

We also treated multiple direct estimates available for each of the five departments as independent in the linear model. If data are missing completely at random, treating correlated data as independent has little influence on the estimation target, but can have strong influence on the variance, so the assumption has consequences. However, we could not implement multiple capture–recapture based estimates of population size proposed in Castledine (1981) due to the lack of sufficient information on overlaps between the surveys. Bao, Raftery, and Reddy (2015) have demonstrated how to estimate population sizes by incorporating data from multiple surveys and other data sources, in a fully Bayesian setup. While we have multiple estimates for each department, all of them are based on RDS and it is not clear how to adapt that approach when working with such estimates. Directly using the individual survey data and the counts from the four sources as an input to the model would be an even more fundamental approach. In addition to accounting for dependence among the four sources, this will also allow us to shift from the two-stage paradigm adapted in this analysis, where the direct estimates were plugged into the extrapolation model, to a unified framework yielding direct and extrapolated estimates simultaneously and ensuring complete propagation of uncertainty. However, incorporating the RDS network into a hierarchical Bayesian area-level model to produce full distributional inference remains a challenging problem.

These are substantial methodological challenges concerning direct estimates that we circumvented. Our central focus was to extrapolate to areas with no data whatsoever using standard direct estimates from the five departments. However, since the extrapolation results depend on the quality of the direct estimates, advancing the statistical theory of RDS based estimates, in conjunction with more data collection, is critical for improving the results.

The small number of areas with direct estimates for MSM has been a major limiting factor in modeling and has contributed to the large prediction uncertainties at some of the areas. Other relevant datasets, like MSM populations in other countries, if available, can be potentially leveraged to borrow strength in parameter estimation. However, care is needed when leveraging data from other countries, as different countries often have entirely different key population dynamics and borrowing strength may not be meaningful. Perhaps, more useful will be data for other associated key populations like FSW, for the same set of areas. The correlation can be exploited in a multivariate setup to improve estimation of both populations. Such a bivariate extrapolation approach will rely on assumptions less extreme than borrowing information across countries.

To our knowledge, we present the first empirically calculated estimates of the numbers of MSM in all areas of Côte d’Ivoire prioritized in the HIV response. Our context of limited centers with measured population size is also not uncommon in the areas of the world where HIV prevalence is the highest given that these settings often also tend to criminalize same-sex practices or at least have significant stigma affecting MSM. In Southern and Eastern Africa, there is often only HIV prevalence data and size estimate data in one or a few urban centers for MSM, though, where studied, the HIV prevention and treatment needs are significant across these countries. Côte d’Ivoire, in West Africa, has one of the larger HIV epidemics in the area, but limited information has been traditionally available on the numbers of MSM and the HIV burden among them. Thus, while our estimates provided here require further validation by supporting data to be collected in additional centers for MSM, where predictions were completed, in the interim, these estimates can support the planning of the scale and content of HIV prevention and treatment programs for MSM in Côte d’Ivoire. Specifically, areas with wide credible intervals should be targeted for future surveys to improve modeling precision. Subsequently, validation and additional data points will highlight the strengths and weaknesses of the current approach and pave the way for modeling improvements.

## Figures and Tables

**Table 1.** Population size estimates (and 95% confidence intervals) of MSM in age group of 18–29 years.

Areas	NGO membership	Service multiplier	Social event	Unique object

Abidjan	3535 (2593, 5550)	2759(2083, 4087)	2334(1669, 3879)	1910(1412, 2947)
	2334(1773, 3415)			
Agboville	1015(807, 1369)		480 (351, 760)	823 (599, 1315)
Bouake	3873 (2536, 8190)	747 (600, 988)	473 (397, 586)	831 (708, 1006)
Gagnoa		947 (628, 1925)	384(287, 581)	555 (409, 860)
Yamoussoukro	1036 (721, 1835)	1688(1038, 4517)	398 (300, 589)	983 (754, 1412)

**Table 2.** Leave-one-out cross validated mean square error for the four models.

Covariates included	MSE_LOOCV_

Log(male population)+ HIV prevalence	3.5 × 10^−3^
Landscan density + HIV prevalence	5.7 × 10^−3^
Population density + HIV prevalence	6.5 × 10^−3^
Density change+ HIV prevalence	6.7 × 10^−3^
Only HIV prevalence	20.0 × 10^−3^

**Table 3.** Posterior median and 95% credible interval for the hierarchical model.

*β*_0_	2.62 (0.04, 5.23)	*μ*	2.55 (1.54, 3.61)
*β*_1_	−0.79 (−1.09, −0.51)	*σ*^2^	0.86 (0.5, 2.29)
*β*_2_	0.63 (−0.07, 1.33)	*ϕ*	7.68(2.71, 9.89)
*τ*^2^	0.65 (0.34, 1.43)		

## Appendix A: RDS Questions for Multiplier Methods

Questions asked of MSM recruited to participant in an RDS survey in order to provide an independent source of data for size estimation in Côte d’Ivoire in 2014

- Unique object: “Did you receive this object before?” [show single object]
- NGO membership: “Are you a member of the NGO Rainbow Plus, or have you ever participated one oftheir activities or even was hit by one of their peer educators?”
  “Are you a member of the NGO Alternative CI, or have you ever participated one of their activities or even was hit by one of their peer educators?”
- Service: “Have you received care at the Clinique de Confiance through the year 2014?”
- Social event: “Have you participated in the social event called “evening GNARA” which took place on Saturday, March 21, 2015 to space Embassy located in the Riviera II?”

## Appendix B: Size Estimate Predictions for All 61 Departments

**Table B1.** Predicted MSM population size, population fraction and HIV prevalence along with credible intervals (within braces). Bold font indicates HIV prevalence for areas where there was data on it (this data was taken from UNAIDS report which did not provide any confidence intervals).

areas	MSM population size	MSM population %	HIV prevalence

Abidjan	1818 (1113, 2941)	0.5 (0.3, 0.8)	**3.6**
Agboville	628 (352, 1134)	6.5 (3.7, 11.8)	**3.1**
Bouake	953 (741, 1218)	1.3 (1.0, 1.7)	**3.1**
Gagnoa	383 (187, 783)	1.6 (0.8, 3.2)	**2.0**
Yamoussoukro	822 (614, 1101)	2.3 (1.7, 3.1)	**3.1**
Abengourou	638 (457, 889)	1.8 (1.3, 2.6)	**2.7**
Agnibilekrou	755 (472, 1218)	4.3 (2.7, 7.0)	**3.2**
Beoumi	476 (305, 746)	3.1 (2.0, 4.8)	**2.5**
Biankouma	356 (176, 721)	2.1 (1.0, 4.2)	**2.0**
Bouafle	591 (377, 922)	1.3 (0.9, 2.1)	**2.5**
Bouna	328 (164, 666)	2.8 (1.4, 5.8)	**2.0**
Dabakala	497 (323, 769)	2.6 (1.7, 4.0)	**2.5**
Daloa	439 (182, 1051)	0.7 (0.3, 1.6)	**1.9**
Danane	421 (216, 816)	1.5 (0.8, 2.9)	**2.1**
Dimbokro	377 (216, 673)	4.1 (2.4, 7.3)	**2.3**
Divo	422 (198, 894)	1.1 (0.5, 2.3)	**2.0**
Guiglo	772 (495, 1214)	4.0 (2.6, 6.3)	**3.2**
Issia	387 (171, 865)	1.1 (0.5, 2.5)	**1.9**
Katiola	1134 (413, 3078)	10.3 (3.7, 27.8)	**4.0**
Korhogo	543 (301, 975)	1.0 (0.6, 1.8)	**2.3**
Lakota	374 (184, 761)	1.7 (0.8, 3.5)	**2.0**
Man	680 (503, 914)	1.9 (1.4, 2.6)	**2.8**
Odienne	261 (110, 630)	2.8 (1.2, 6.7)	**1.7**
Oume	540 (354, 829)	1.9 (1.2, 2.9)	**2.5**
Sakassou	262 (111, 634)	2.7 (1.1, 6.6)	**1.7**
Sassandra	1037 (647, 1684)	3.2 (2.0, 5.2)	**3.5**
Seguela	328 (141, 755)	1.6 (0.7, 3.6)	**1.8**
Sinfra	525 (343, 806)	2.1 (1.4, 3.2)	**2.5**
Toumodi	488 (313, 763)	3.8 (2.4, 5.9)	**2.6**
Vavoua	278 (80, 948)	0.6 (0.2, 2.2)	**1.3**
Zuenoula	214 (57, 783)	1.0 (0.3, 3.5)	**1.1**
Bettie	525 (179, 2025)	8.9 (3.0, 34.4)	3.0 (1.6, 4.3)
Blolequin	597 (204, 1859)	4.1 (1.4, 12.7)	2.9 (1.6, 4.2)
Bocanda	473 (153, 1342)	3.8(1.2, 10.8)	2.6 (1.2, 3.9)
Botro	612 (218, 2234)	7.9 (2.8, 29.0)	3.1 (1.8, 4.5)
Didievi	434 (136, 1345)	4.9 (1.5, 15.2)	2.5 (1.2, 3.9)
Dikodougou	415 (129, 1253)	5.3 (1.6, 15.9)	2.5 (1.2, 3.8)
Djekanou	261 (81, 890)	9.5 (3.0, 32.4)	2.6 (1.3, 3.9)
Doropo	328 (89, 945)	5.0 (1.4, 14.4)	2.2 (0.8, 3.5)
Fresco	527 (180, 1695)	4.9 (1.7, 15.8)	2.8 (1.4, 4.1)
Gbeleban	111 (28, 344)	6.0 (1.5, 18.5)	1.9 (0.5, 3.2)
Guitry	474 (140, 1309)	3.0 (0.9, 8.4)	2.5 (1.2, 3.8)
Kani	304 (79, 849)	3.9 (1.0, 11.0)	2.0 (0.7, 3.3)
Kouassi-kouassikro	263 (82, 921)	9.3 (2.9, 32.4)	2.6 (1.2, 3.9)
M’bengue	363 (103, 999)	4.1 (1.2, 11.3)	2.3 (1.0, 3.6)
Madinani	255 (66, 776)	6.4 (1.7, 19.4)	2.0 (0.6, 3.3)
Nassian	347 (102, 1150)	7.6 (2.2, 25.3)	2.4 (1.1, 3.7)
Niakaramandougou	636 (229, 1990)	4.6 (1.7, 14.4)	3.0 (1.7, 4.3)
Samatiguila	106 (25, 327)	5.8 (1.4, 18.1)	1.8 (0.5, 3.2)
Seguelon	166 (43, 514)	6.3 (1.7, 19.6)	2.0 (0.6, 3.3)
Sikensi	547 (195, 1838)	6.7 (2.4, 22.5)	2.9 (1.6, 4.3)
Sinematiali	363 (110, 1114)	6.4 (2.0, 19.7)	2.4 (1.1, 3.7)
Sipilou	285 (76, 873)	6.3 (1.7, 19.2)	2.1 (0.7, 3.4)
Taabo	371 (116, 1122)	6.2 (1.9, 18.8)	2.4 (1.1, 3.7)
Tai	567 (193, 1822)	5.0 (1.7, 16.2)	2.9 (1.5, 4.2)
Tehini	310 (88, 996)	7.5 (2.1, 24.1)	2.2 (0.9, 3.6)
Tiassale	550 (178, 1557)	2.8 (0.9, 7.9)	2.7 (1.3, 4.0)
Tiebissou	443 (139, 1277)	4.4 (1.4, 12.7)	2.5 (1.2, 3.8)
Toulepleu	459 (148, 1554)	7.4 (2.4, 24.9)	2.7 (1.4, 4.1)
Zouan-hounien	511 (157, 1350)	2.6 (0.8, 6.8)	2.5 (1.2, 3.8)
Zoukougbeu	342 (87, 905)	2.8 (0.7, 7.3)	2.0 (0.7, 3.4)
